# The role of product development practices on new product performance: Evidence from Nigeria's financial services providers

**DOI:** 10.1016/j.techfore.2020.120470

**Published:** 2021-03

**Authors:** Nkemdilim Iheanachor, Immanuel Ovemeso Umukoro, Olayinka David-West

**Affiliations:** a Strategy and International Business Group of Lagos Business School, Pan-Atlantic University, Lagos, Nigeria; b Information Systems & Digital Business Transformation, Lagos Business School, Pan-Atlantic University, Lagos, Nigeria; c Professor of Information Systems, Lagos Business School, Pan-Atlantic University, Lagos, Nigeria

**Keywords:** Product development, Product performance, Financial service providers, Emerging markets

## Abstract

This study investigates the impact of product development practices on the performance of new financial products and services through the analysis of ten in-depth case studies. We argue that weak product development practices negatively affect product performance. This study finds that in Nigeria, new financial product performance is suboptimal because of poor product development practices. This study further shows that when poor execution follows inadequate product development practices, the likelihood of product failure increases, as evidenced by poor product performance and low adoption. The processes adopted in the development of financial services affect the adoption, use, and overall penetration of the product in the target market. Therefore, this study suggests that the management team of various financial service providers invest in developing sound product development practices in the actualization of their goals of increasing the adoption and use of their products.

## Highlights

• Impact of product development practices on the performance of newly launched products.
• When key product development practices are not well implemented the likelihood of product failure increases.
• Development of financial service products affect adoption, use and product penetration.
• Management teams of various financial service providers should invest in developing sound product development practices.

## 1 Introduction

In emerging markets, financial services innovation (whether in products or business models) is critical to a provider's sustainability and competitiveness (David-West et al., (2019)). Technological innovation has disrupted the financial services sector by establishing novel ways of creating and delivering value to customers (Hine and Greenaway, 2016; Li, 2016; Madeira, 2016). This warrants additional investments in information technology (IT) assets, resources and capabilities. As IT investments increase, customer needs are also becoming more diverse. Financial service providers (FSPs) must also strengthen their processes to meet and surpass the needs of their customers who are the most influential yet volatile stakeholders (De Oliveira and Rabechini, 2019; Kim et al., 2018; Ouma et al., 2017; Pollari, 2016).

Understanding consumer pain points remains a critical driver in creating and delivering a compelling customer value proposition that meets their diverse financial service needs. David-West et al., 2019 report that customer value propositions (CVPs) such as affordability, accessibility, ease of use, service reliability and security characterize financial products and services. FSPs need these CVPs to acquire and retain customers. To achieve and sustain these attributes in their products, FSPs must continually review their customer value propositions and product fit amidst changing consumer behaviours. The continuous review of product development practices is one such approach. This is undoubtedly true, especially in an era where new non-bank financial technology (Fintech) firms are challenging the incumbents and disrupting the financial services space with new value propositions.

Financial product development requires the commitment of critical resources and an understanding of customer needs, characteristics and behaviours to gain the adoption, customer satisfaction (Brockmann, 2017; Laukkanen, 2016; Park and Koh, 2017), and continuous use of financial services (David-West et al., 2019b). Well-developed financial products yield benefits such as improved market shares, higher profits, returns on equity, customer loyalty, and long-term survival (Albers et al., 2018; Lalic et al., 2018; Mikkola, 2018). For new products to be successful, organizations must ensure that product development processes such as ideation, prototyping, testing and launching must be carefully and systematically executed (Roy et al., 2017; Yoon and Rim, 2018; Claudy et al., 2016). Beyond the value proposition, pilot and launch strategies that FSPs adopt can also affect product acceptance. There is, therefore, a need to be innovative in the product development process. Such processes require a competent team, efficient operational processes, and a strategy for managing emerging product risks.

The spike in financial product failure in emerging economies is caused by a lack of adherence to good product development practices (Albers et al., 2018; Lalic et., 2018; Mikkola, 2018). For instance, low financial inclusion rates in Nigeria can be attributed to poor product adoption resulting from poor product-customer fit and other exclusion enablers. Evidence of failed financial products abounds in Asia (Kim et al., 2016) and Africa (Karabag, 2019; Šoltés et al., 2017; Gumel, 2017). These products often fail to address customers’ pain points. Likewise, product failures have significant impacts on customers, employees, profitability, market share, brand equity, investors, and the economy at large. Such failures can erode consumers’ trust in a brand and could be capitalized upon by rival firms. This study investigates the impact of product development practices on the performance of newly launched products by financial service providers by relying on evidence provided by ten case studies of purposively sampled FSPs.

The goal of product development practices is to meet consumer needs while keeping a focus on profitability and business sustainability. Products are one dimension of competition in the financial services space. The practices that produce the products must receive adequate attention. They are vital to improving the quality of services to promote customer adoption, consumer satisfaction, customer retention, profitability and long-term sustainability.

### 1.1 Rationale

The concept of product development has been widely discussed in the literature, especially in innovation management research. However, the analysis of the literature on product development practices shows that most references and case studies are in the manufacturing (Akroush and Awwad, 2018; Chang and Taylor, 2016; Vinayak and Kodali, 2014), telecom (Namusonge et al., 2017), aviation (Naghi Ganji et al., 2017) and transportation sectors. Studies on product development practices of financial service providers remain sparse, especially those from an emerging economy perspective. This phenomenon is essential given the low level of adoption of financial services in Nigeria, resulting in a low financial inclusion rate despite the broad spectrum of financial products and services offered by Nigerian FSPs. Where financial products exist, their adoption and utility are usually low. The adoption of financial services (used interchangeably with financial products) is a significant phenomenon as it plays a critical role in enabling Nigeria to attain its financial inclusion goals of 80% by 2020. Despite the high number of bank and non-bank FSPs in Nigeria, approximately 36.8% (36.6 million) of the adult population remains financially excluded. Additionally, 14.6% (14.6 million) of adults are underserved. The underserved segment resorts to the use of informal channels and services (EFInA, 2018), which could be costly and risky.

The challenge of financial inclusion in Nigeria and its consequent negative impact on economic development continues to attract the attention of scholars and governments at all levels. Several factors are responsible for this low level of adoption. While a demand-side approach may amplify the voice of financial services consumers, a supply-side approach provides a broader perspective on the processes through which FSPs conceptualize and develop products. This can offer insight into how product development practices may contribute to low financial service adoption and, by extension, low financial inclusion. Financial exclusion can be due to lack of product-customer fit, often resulting from flawed research and development efforts. This can be because of insufficient business case analysis, ineffective market segmentation, the absence of product prototype development, and insufficient testing and refinement, among other causes.

Specifically, the study examines the following:

1 The nature of product development practices (PDPs) among financial services providers (FSPs) in Nigeria
2 The impact of product development processes on new product performance in Nigeria
3 How the risk management strategies of financial services providers affect the performance of the new products of financial service providers

### 1.2 Research questions

The following research questions guided this study:

1 How do Nigerian FSPs undertake product development?
2 To what extent do best product development processes guide Nigerian FSPs in their new product development efforts?
3 What is the role of product development processes in the performance of new products developed by Nigerian FSPs?

We organize the rest of this paper as follows. The section after this introduction provides a brief review of the related literature on product development practices. It aims to explore the relevant literature and outline extant theories within the context of product development practices. The method section follows the literature review. The results and discussion sections are next; followed by a conclusion, recommendations, implications for practice and directions for further studies.

## 2 Literature review

### 2.1 Product development and product development practices

**Fig. 1:**
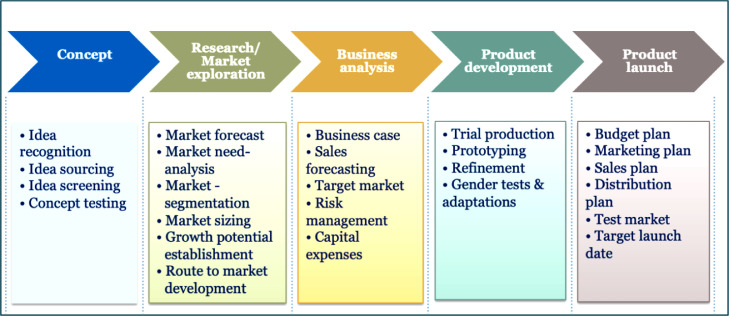
Product development framework (*Source: Author's representation*).

The term product development or new product development refers to the transformation process of a market opportunity and a set of assumptions regarding product technology into a product accessible to the market (Chang and Taylor, 2016). It is a process that leads to introducing new products into a market as a response to a market opportunity by logically combining a set of activities. Product development practices (PDPs) are a defined set of tasks, steps and phases that describe the standards by which a company repetitively converts embryonic ideas into sellable products or services (Kahn, 2004). They are firm practices that translate into the development and launch of new products as a response to new market opportunities. PDPs are "success drivers of new product development efforts" (Troy et al., 2008, p. 136) because when properly implemented, they can positively impact an organization's market share, profitability and long-term survival. PDPs include practices that help business organizations arrive at quality and viable products that meet market needs and can capture value for the organization while creating value for customers (see Fig. 1). The concept impacts three broad aspects of organizational success: operational, financial, and marketing performance.

Nguyen et al. (2018) note that practices such as the development of product programmes, research and development (R&D) and innovation can translate into the success of a new product. There are two categories of PDPs – process speed and integrative practices.

■ *Process Speed:* This refers to the compression of activities (Kiss and Barr, 2017) versus traditional sequential new product development practices. Process speed could be agile development, early feedback or late decision-making product development practices for accelerating the speed of the product development process. *Agile development* is characterized by rapid development iterations used to gain feedback combined with overlapping processes where the next iteration begins before the current iteration finishes (Haidar et al., 2017; Mohan et al., 2010). Agile development contrasts with the traditional waterfall development methodology that focuses on preparing a complete and detailed design specification before the execution phase begins (Guntamukkala et al., 2006; Roems, 2017). *Early feedback* refers to regularly gathering feedback from multiple constituents at the earliest stages of the product development process (Lakemond et al., 2013; Thomas, 2014; Narasimhan et al., 2006). *Late decision* making is a process in which product concepts, capabilities and designs are not finalized until the last phases of the development process (Buganza et al., 2010, 2009). Late decision-making contrasts with the traditional stage-gate style processes where product development is in a sequential structure of decision gates (Kahn, 2004). At each decision gate, a facet of the product is agreed upon and frozen before moving to the next gate.
■ *Integrative* P*ractices:* These are processes used by the organization to regenerate its knowledge base (Eisenhardt and Martin, 2000; Kogut and Zander, 1992; Marsh and Stock, 2006). They include foundational customers and supplier participation. *Foundational customers* are customer representatives who participate in the new product development process in a manner that helps shape the requirement analysis (Carbonell et al., 2009; Gatignon and Xuereb, 1997) for new product development. In-depth requirement analysis of market realities is critical for the success of financial products. Validating initial market assumptions requires engaging customers that can provide *a near real-life* input to the requirement analysis and initial product design stages. *Supplier participation* refers to the various roles that suppliers play in the product development process. It ranges from merely delivering parts based on a specification to substantial involvement in the design process (Ragatz et al., 2002; Gatignon and Xuereb, 1997; Cusumano and Takeishi, 1991). Suppliers are a critical category of stakeholders in the product development process. The interface with customers provides useful feedback on customer buying and consumption behavior. Lau, 2011 also state that supplier involvement and inter-functional integration can also eliminate barriers that lead to new product failure.

### 2.2 Product performance and performance measures

Product performance refers to how well a product performs across defined measurement indicators. The indicators could be how product development promotes customer attraction (market share) and retention, revenues and net profit, brand equity, customer satisfaction and feedback, among other indicators (Namusonge et al., 2017; Ganeshkumar and Nambirajan, 2013; Schilling and Hill, 2005). Product performance reflects the financial and market performance of a firm's new or existing product (Najafi-Tavani et al. (2016)). Product performance measures or outcomes are the actual performance of a product against the expected level of performance. They are indicators that measure changes that the firm needs to manage the transition towards defined goals. In examining how firms benefit from new product development, we can broadly categorize product performance into four distinct dimensions:

■ *Profitability (financial) Performance*: Financial performance is the degree to which the product exceeds or falls short of the expected profitability level (Cooper, 2019). The profitability dimension includes both the level of profit and profit objectives. On average, the level of profit is scored relatively higher than the score for profit against the objective. It, therefore, implies that firms expect a higher level of profit from introducing new products.
■ *Sales and Market Performance*: Market performance is the extent to which the product exceeds or falls short of achieving market expectations (Cooper, 2019). Sales performance illustrates the performance of new products and comprises measures of performance relative to sales objectives, and measures of total sales. Sales performance is growth in sales against the aim.
■ *Customer Satisfaction***:** This is the level of the purchaser's affective response. It is an assessment indicator of how well financial services perform. High adoption is due to customer satisfaction with the product. However, Umukoro and Tiamiyu (2017) warn that in the context of e-services, the absence of better alternatives may increase use without necessarily translating into customer satisfaction. One way of ensuring customer satisfaction is for FSPs to define a product value proposition in ways that address customer needs (David-West et al., 2019b, 2017; Mbiti and Weil, 2013).
■ *Enhanced Opportunities*: These are the gains of product development practices to the firm as an entity rather than solely accruing to the product. This factor illustrates the long-term benefits that can be derived from introducing a new product. Repositioning the firm, creating a new market, and platforms for the introduction of additional new products or new product features increase the potential for long-term prosperity.

### 2.3 Product development practices and product performance (success)

Schilling and Hill 1998 suggest that for a firm to be successful at new product development, it must simultaneously meet two critical objectives: maximizing profits through customer needs and minimizing the time to market. While these objectives often pose conflicting demands on a firm, there is a growing body of evidence that a firm may adopt strategies to meet these objectives successfully. Successful companies are known for articulating their strategic plan and leveraging their R&D portfolio to achieve a fit between their new product development goals and their current resources and competencies. Namusonge et al (2017) posits that strategic product development practices have a positive and significant influence on financial performance.

Many products fail too quickly because of weak market analysis, poor design (weak products), regulatory risks, weak and unvalidated market assumptions, and late arrival to market, among other reasons. Ateke et al. (2015) note that firms can also measure the performance of a new product in terms of the levels of customer adoption and satisfaction, the profitability of the new product, and how long the product survives competition from rival products. It is important to note that how well a new product performs in terms of financial performance, customer adoption, growth in market share, and customer satisfaction is a function of the product development practices that the firm adopts (Nguyen et al., 2018).

Successful products need a strong product development team to conduct practices that foster the success of developed products (Naghi Ganji et al., 2017). Product managers must, therefore, understand the business impacts of product development decisions and the need to have the right product development practices in place (Nguyen et al., 2018). Often, product development managers are quick to isolate the causes of poor product performance and may tackle them individually. However, a combination of these factors may exist. Cassia et al. (2012) argue that new products and ideas fail because they lack structured product development processes or practices. The absence of efficient product development practices such as risk management, product development strategy, research and development and other practices can lead to weak products (Almeida and Miguel, 2007). This can lead to poor market and requirement analysis resulting to products that do not align with customer needs.

### 2.4 Product development practices include the following

■ *Research and Development:* Product innovation begins with an understanding of a need and how well to solve the need. Frankort (2016) reports that knowledge acquisition through R&D is positively associated with product performance both in terms of product breadth and market performance. Many organizations, including those in the financial service sector, are engaging in R&D to be more deliberate in the products they introduce. Organizations such as Google, Apple, and Microsoft take R&D further and include the establishment of research, development and innovation labs to aid their product development efforts. With greater involvement in R&D, products perform better in terms of profitability, adoption, and usage (Cuervo-Cazurra et al., 2018; Santoro et al., 2017; Homburg et al., 2017; ). Considering this, we argue the following:

Proposition 1: Research and development practices enhance new financial product performance.

■ *Well-established or Structured Product Development Processes:* Product development involves a logical implementation of a set of activities (Chang and Taylor, 2016; Kahn, 2004). Good product development practices include well-thought-out processes that follow a product development methodology. Although many methodologies exist, certain features characterize them. For instance, product requirement analysis is a necessary process or practice that must be undertaken to understand customer and market dynamics and validate initial market assumptions. A well-structured product development process also considers critical activities in the product development life cycle. These activities include customer empathy and ideation, the determination of a business case, design and prototyping, testing and launch, and product performance assessment. Given these assumptions, we argue the following:

Proposition 2: Structured product development processes contribute to the success of new financial products.

■ *Product Development Strategy:* The product strategy is a plan that focuses on the product efforts directed towards achieving business goals. It is a set of actions in a sequence explaining why this is the right approach. Poor products can also result from a poor product strategy, given that the strategy determines the products’ impact and performance. Product development efforts become aimless without a defined strategy, just as a strategy is useless without execution. The product development strategy helps contextualize the problem that the product *will solve, for whom, when and where* should a new financial product be introduced. A well-articulated product strategy helps a firm to assess how its product development capabilities match the market opportunities. Such capabilities may include leadership, functional and technical skills. Where existing capabilities are inadequate for exploring market opportunities, the firm must strategize on how to play, where to play, and when to play to win (Ogechie, 2018). This can significantly increase the chances that the proposed product will perform well when finally developed. Consequently, we argue the following:

Proposition 3: The existence of a product development strategy enhances the success (performance) of new financial products.

■ *Risk Management:* New product development efforts often face risks that, when not well managed, may lead to product failure; and sometimes, product development efforts may not even materialize. Risks differ across different organizations and product lines. While the risk profiles for different financial products may not be identical, it is essential to identify where on the spectrum a company wants to be to plan risk mitigation measures. Consequently, we argue the following:

Proposition 4: Risk management practices can enhance the performance of new financial products.

### 2.5 Theoretical foundation and research framework

The dynamic capabilities (DCs) theory extends the well-established resource-based view (RBV) theory. The dynamic capabilities theory emphasizes the ability of a firm to integrate, develop and reinvigorate its internal capacity to address challenges arising from rapidly changing business environments (Teece et al., 1997, p. 516). From the above definition, we can infer that DCs promote continuous change and the configuration of the productive resources of a firm to adapt better to the environment.

The literature provides empirical evidence that suggests that the management of various competitive organizations invests in product development practices as a strategic solution for long-term survival in some dynamic environments (e.g., Pavlou and El Sawy, 2011; Schilke, 2014). Regular product development practices (PDPs) and product introduction require a variety of activities that are the driving forces to regenerate and renew the routines and competitors' strategies of a firm, ensuring environmental adaptation in various industries (Helfat and Winter, 2011). The DC theory provides the underpinning for this case study on the product development practices of financial services as it helps to explain how financial service providers develop and integrate assets, resources and capabilities for new product development as a response to the needs of a changing business environment.

**Fig. 2:**
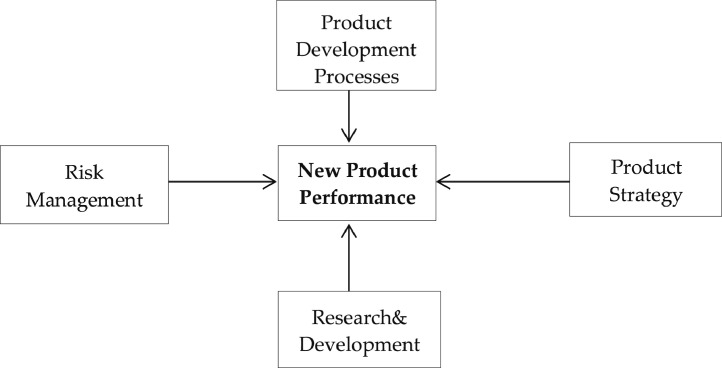
Research framework.

Fig. 2 shows four critical product development practices – product development processes, risk management, research and development, and product strategy – as factors that affect the performance of new financial products.

## 3 Methodology

This paper is an exploratory study. It adopts a qualitative method using multiple case studies of eight (8) financial services providers to investigate the product development practices and the effects of these practices on new product performance. Case studies provide very engaging and rich explorations of a project as it develops in a real-world setting (Berkowitz, 1997). The study uses a cross-case analysis given its robustness for analysis and synthesis of data across multiple sources, unlike the individual or intra-case analysis approach that restricts the analysis to a single case (Cruzes et al., 2015; Miles et al., 2013; Berkowitz, 1997; Mahoney, 1997;). We collected data on product development practices using semi-structured interviews.

Theories and concepts from the existing literature were identified (see Table 1) and used in the development of question items for the interview guide. Pertinent questions were framed and validated for each of the constructs. We derived the questions from an item generation process while incorporating themes, noting patterns, seeing plausibility, clustering, making metaphors, counting, contrasting/comparing and partitioning variables (David-West et al., 2018; Miles et al., 2013). The final instrument is a semi-structured interview developed from the validation of questions conducted through several iterations of expert review. Convenient and purposive sampling of FSPs (cases) was conducted to select the respondents who were within reach. The purposiveness of the sampling approach ensures that the data were collected from product development managers or senior team members (see Table 1) at FSP headquarters, where product development and decision-making processes are located.

**Table. 1:** Profile of interviewees.

FSPs	No. of Interviewees	Deparments/Functional Areas
FSP1	1	Operations
FSP2	1	Operations
FSP3	1	Transaction & Electronic Banking
FSP4	2	Management, Operations, and E-Products
FSP5	1	Alternative Delivery Channel
FSP6	4	Retail & SME Banking, and Mass Market, and Agency Banking
FSP7	1	Digital Banking
FSP8	3	HR and Admin, Operations, and Enterprise Risk Management
FSP9	1	Marketing
FSP10	1	Strategy and Communication

*All respondents interviewed are of the managerial cadre.

Table 1 highlights the sampling profiles used in the study. Embedded research ethics protocols guided the practices used in seeking formal participation consent and session recordings. An a priori list of codes guided the coding and analysis of interview transcripts. The hierarchical code structure from the a priori list of codes was replicated in an Nvivo QDA environment (David-West et al., 2018).

## 4 Results

### 4.1 Demographic characteristics of the interviewed FSPs

In Table 2, we present the main characteristics of these ten FSPs. For the reason of confidentiality, financial services providers have been anonymized in the table.

**Table. 2:** Main features of selected FSPs for multiple case studies.

***FSPs***	***Institution Type***	***Types of Digital Financial Services (DFSs) FSPs provided***
FSP1	Microfinance Institution (MFI)	Loans, micro and nano loans, clean energy financing, asset financing, SME loans, and micro-savings
FSP2	Bank-led Mobile Money Operator (MMO)	Savings products and credit products
FSP3	Deposit Money Bank (DMB)	E-payment bills, school solutions, collections, and trade
FSP4	PSSP Aggregator, Switch & Super-Agent	E-wallet, bank-IT, corporate pay, web connect, switch-it, pay outlet, ATM cardless cash, and mobile top-ups
FSP5	Microfinance Bank (MFB)	Loans, savings accounts, savings plan accounts, regular savings accounts, festival savings, social impact deposits, term deposit savings accounts, voluntary savings accounts, individual savings, savings, housing, and clean energy
FSP6	Deposit Money Bank (DMB)	Letters of credit, unity pay, unity collect, and unity remits
FSP7	Micro Insurance	Investment products (money market funds, equity income funds, education trusts, employment investment schemes, and customized portfolios), insurance product (life insurance, motor insurance and travel insurance), goods in transit, fire and other peril, money insurance, group life insurance, savings and investment
FSP8	Non-Bank led Mobile Money Operator (MMO)	Corporate e-products, treasury products, and trade products
FSP9	Non-Bank led Mobile Money Operator (MMO)	Corporate, retail, FetsTraq, and PTSP solutions
FSP10	Micro Pension	Retirement savings accounts (RSAs)

Many of the FSPs interviewed mentioned that several financial services were developed within the last five years. As noted earlier, a plethora of financial products has always characterized the Nigerian financial service market. However, many of these products underperform. The products shown in Table 2 can be broadly grouped into savings, credits, insurance, pensions, utility and bill payment, corporate banking, SME banking, and remittance products.

### 4.2 Product development practices and level of implementation by FSPs

Table 3 summarises the different product development practices of FSPs and the FSPs' performance levels on each of those practices reported. The dominant product development practices of FSPs include R&D, product design and prototyping, risk management, product performance measurement, strategy formulation and execution, and impact measurement. As shown in Table 3, all FSPs reported engaging in all the product development practices that were identified. In attempting to assess how product development practices affect new product performance, the study also assessed the level of implementation of these product development practices. The results are presented in Table 3.

**Table. 3:** Product Development Practices (PDPs) and FSPs Scorecard on each PDP.

The results presented in Table 3 show that the level of execution of product development practices varies across different FSPs. However, similar patterns exist among FSPs with similar assets, resources, and capabilities (ARCs). The results are discussed in the following section.

## 5 Discussion

### 5.1 Nature of product development practices among Nigerian FSPs

We assess the product development practices conducted by the different financial service providers to understand the differences and similarities and the attendant's reasons for the level of PDPs conducted.

• *Product Development Practices among Mobile Money Operators (MMOs):* Mobile money operators (FSPs 2, 4, 8, & 9) performed low or moderate across the PDP measures. These institutions primarily provide payment services through mobile devices. They rely mainly on the quality of mobile network connectivity and the spread of their agents across different locations of interest. MMOs typically offer higher-priced services because of the high service charges they incur from different partners who provide the service delivery channels. Manpower costs are high, which limits the product development capabilities of these providers. The assessed risk is average as payment services do not involve the extension of credit. MMOs have a significant need for high-quality talent, which is expensive and scarce. Market sizing and product viability assessments are non-existent. Market and occupational segmentation are also not visible, although product development and deployment are driven by profitability assessments. Low levels of ideation and market testing are observed.
• *Product Development Practices among Pension and Insurance Providers:* Pension and insurance providers scored low in R&D, customer empathy and ideation, business case determination, product design, prototyping, testing and launch, and product impact assessment, although both FSPs (7 and 10) scored moderately in their risk assessment practices. These institutions primarily focus on providing affordable retirement savings to working individuals to make them financially secure and independent in their old age. Most product offerings are homogenous, and the product design exists only within the regulatory boundaries defined by PENCOM.

These regulatory safeguards are heavily skewed towards risk management with little incentives to potential savers compared with other FSPs that offer them credit. They mostly engage in product adaptation rather than developing new products to meet the needs of banked and served consumers. Risk management here is very strong while leveraging the risk management capabilities of the parent pension companies. Market sizing and product viability assessments are very scant. They do not have products that are gender specific, but they have products that speak to the general needs of the consumers outside the pension net. We also observed a low level of prototyping and product testing among pension and insurance providers.

• *Product Development Practices among Microfinance Banks/Institutions:* These institutions (FSPs 1 and 5) also exhibited similar product development practices. While they score moderate across several PDPs, more attention is given to business case analysis, product design, and prototyping. These institutions are part of the FSP segment in Nigeria that customizes services with ethical lending practices in the form of small business loans to unemployed, low-income individuals. These people would otherwise have limited or no access to other financial products, especially in semi-urban and rural areas. There are no procedures for how to track product performance. The most popular products are near-identical. It takes from two months to two years for them to launch new products. The prototyping and actual product development process are also unclear. MFBs do not have products that are gender specific, and they have fewer cost reduction initiatives in terms of interest rates. MFBs identify the need for new products through agent networks, organic movement, and interviews (FGDs) that provide them with an understanding of the dynamics of consumer behaviours and locations.
• *Product Development Practices among Deposit Money (Commercial) Banks:* Although deposit money banks (FSPs 3 & 6) are not very similar in product development practices, none of the DMBs scored high in any of the product development practices. DMBs have well-resourced product development teams that come from diverse functional areas in the organization. Providers in this area conduct more product adaptation and offer the same product to virtually all consumers. Minimal customization occurs. These factors lead to low product adoption; and where adoption exists, customer attrition is high. Risk assessment is high, and this could reduce adoption. Market sizing and product viability assessments are very scant. Market and occupational segmentation are shallow as providers rely more on customer feedback to gage market needs, some of which are unstructured or inaccurate. For DMBs, product development is better resourced. Product testing and roll-out can take an average of 2 to 3 months with regulatory approval not serving as a major impediment. A low level of ideation is widespread and could be the reason for the product homogeneity that is observed among DMBs.

### 5.2 Product development practices and product performance

Proposition 1: Research and Development Practices and Product Performance

In the first proposition, we argue that R&D enhances new financial service performance. All FSPs studied scored low or average on R&D efforts. The findings of this study suggest that poor R&D practice is one reason financial services fail in Nigeria. For instance, a respondent stated the following:

"…our research and development are mainly on market research to map out where the unbanked and under-banked customers are located. That way, our products can be more targeted in our marketing efforts."

This is not an R&D effort that defines the product development direction, but rather it is research that helps the marketing of the product. Beyond demographic profiling, R&D entails behavioural and psychometric profiling of the target market segments to understand their economic lives and the reasons for their behaviours. R&D efforts that consider human-centred design approaches can unravel the various consumer archetypes across various demographic profiles. It is in this stage that the team empathizes with the customer to ideate and refine the value proposition. A good R&D effort provides a clear picture of the problem and potential solutions. A respondent notes thus:

"…I have been involved in some ideation where we have to engage women in some market places to ask them what are the pain points, what are the things that we need to know. We are trying to build a product that will help them to save more and trying to attach some new product to it but we found that what we were thinking about was not what they even wanted and that led to the introduction of a new product to them."

Grimpe et al. (2017) note that innovative product design, packaging, pricing, and promotion that are rooted in R&D can significantly drive the performance of new products. Processes driven by R&D can guide the product development team during the design, prototyping, and testing phases. We conclude that the low level of research and development among FSPs is a driver of the poor performance of new financial services (products) in the Nigerian market.

Another respondent states the following:

"The number of unbanked and under-banked adults in Nigeria is about 50 percent of the adult population. We aim to reach those in places that no one is interested in."

Products developed with this mindset will fail. While the market may exist, not all of it is addressable. Serving a dynamic market such as Nigeria requires quality R&D effort. D'Este et al. (2016) stress that there is clear evidence that firms' knowledge creation capacities, especially internal R&D activities, are decisive for their product performance.

Proposition 2: Product Development Processes and Product Performance

We argue for structured product development processes. They include customer empathy, ideation, concept testing and refinement, requirement analysis and market validation, prototyping, piloting, product lunch and performance assessment. They contribute to the success of new financial products. The data show that FSPs scored low on each product development process except on indicators such as business case determination, design and prototyping, and ideation where a few FSPs scored high (see Table 3). Many FSPs deploy homogenous products to consumers without following a rigorous product development process guided by an established product development methodology. Some FSPs do not conduct customization that ensures that the products meet the needs of the diverse mass market they aim to serve. Homogenous products do not address diverse customer needs and may lead to poor product performance. One of the senior executives interviewed remarked the following:

"…we trust our product development team because they come with a wealth of experience having worked in the manufacturing industry for years. They understand how to carry out product development, and we give them full support once they can show the profitability of the product."

The products that meet the needs of this market may vary across demographic groups and would require understanding customer needs, defining product requirements and business cases, and scenario planning. Good product development practices require product teams to be methodological (Kauffman et al., 2015; Orbach and Fruchter, 2011). These methodological approaches can help in developing products in a manner that considers different customer archetypes across different demographic profiles. This helps to achieve well-defined use cases and customer-centric products with guaranteed wide adoption and profitability. Well-defined and executed product development processes can also help reduce product failures (D'Este et al., 2016) as product development teams can run more iterations of prototypes, allowing for quality checks and determining the product's desirability, viability and feasibility. We conclude that the absence of or a poor implementation of these processes can translate into product failure.

Proposition 3: Product Development Strategy and Product Development Performance

Here, we argue that the availability of a product development strategy enhances the success (performance) of new financial products, given that strategy is required to serve a market efficiently and profitably. Grimpe et al. (2017) posit that a well-developed strategy is critical to successfully taking a product to the market as it considers market forces, especially the threat of substitute products, barriers to entry and other forces that may lower product performance. Product strategies are unique to specific products. The route-to-market (RTM) also differs across locations and demographics. The findings also show that all the FSPs scored low on the product strategy indicator. What many FSPs treat as a product strategy are marketing plans detailing how they will undertake branding and advertising. A respondent notes as follows:

*"…sometimes we have partnerships to deploy some products, so we have to work with banks, like I said pension companies, sometimes we . . …there is a small scale pilot which is with the internal customers, which is me and my colleagues so we are the internal customers, and then expanded retail team, who are the guys who manage the retail product on the field and then we also sample a number of our key agent for a soft life deployment before giving out the products to the customers in general…"*

Product pricing is the responsibility of the finance department, just as operations are an HR concern. There was no strategy document showing how the product will translate into the realization of the overall organization vision. The absence of this has resulted in poor product performance measurement. Drawing from Danneels's (2002) first- and second-order competencies, we can explain that a product strategy helps product managers and teams build marketing innovations that help in aligning new product performance metrics to the overall organizational goal. The absence of this can negatively affect product performance as there are likely to be undefined indicators or approaches for measuring product performance or for taking appropriate corrective measures.

Proposition 4: Risk Management Practices and Product Performance

Proposition four argues that risk management practices can enhance the performance of new financial products. The study observes that the risk management practices in product management are suboptimal among Nigerian FSPs. Few FSPs (mainly MMOs), however, prioritized risk. An MMO representative stated as follows:

"Like I said at the product conceptualization and development, all the functionary units including risk are involved. So, all the risk exposure deliberations are handled at that stage. We have what we call the product papers…in that document every aspect of that product is articulated and documented including the risk mitigants."

A key area where risk is dominant is the platform and not risks relating to product development. FSPs are more concerned with managing risks that are associated with the security of their platforms while neglecting risks that may arise from non-approval by regulators. Such risks, when poorly managed, may lead to products not making it to the hands of the target user groups because of non-approval from regulatory bodies.

## 6 Conclusion

New financial products in Nigeria struggle to perform well because of poor product development practices. Although the financial service sector has grown over the years with an improved regulatory environment, this study shows that product development practices seeking to guarantee new product success are poorly implemented. The resultant effects are poor product performance and low adoption. The processes adopted in the development of financial services affect the adoption, use, and overall penetration of the product in the marketplace. Financial inclusion rates will therefore remain low if the adoption and use of financial services remain low.

Evidence suggests that several financial services are inappropriately designed and unsuited to the needs of the diverse segments of unbanked and underbanked Nigerians. Additionally, there is an overestimation of the market size owing to lack of adequate market research and R&D, which can cause products to not meet financial projections. Products also fail because of poor product designs stemming from inadequate requirement analysis and a lack of well-designed and tested prototypes before products are launched. This can also translate to wrongly positioned, priced, or advertised products that underperform in terms of adoption and profitability. One reason highlighted by this study is that the different FSPs have insufficient product development skills. Most times, product development teams comprise software engineers and those with a manufacturing background whereas financial products are service-oriented. The absence of a skilled and well diversified team can translate to high development costs, which may lead to unprofitable products (Olson et al., 2001).

Addressing these concerns requires FSPs to reconfigure their product development teams. The teams must possess the capabilities needed for the development of quality financial services (products) that meet validated market assumptions. With the right set of product development capabilities, poor product performance can be closed using industry-wide market research that incorporates human-centred design (HCD) and design-thinking techniques. These can lead to the development of financial services that are customer-centric and widely adopted, trusted by consumers, and compliant with market regulations. Additionally, market knowledge breadth that flows from rigorous R&D can help firms transform novel ideas into new products, thereby intensifying product performance (Jin et al., 2019)

Good R&D practices such as the adoption of approaches such as human-centred design can help product managers and the broader product development teams to provide answers that validate initial market assumptions. These could be assumptions on consumer needs, buying behaviour, market share, rival firms, and other prevailing market conditions. These methodologies can help answer questions such as the following: 1) What non-existing value is being proposed by the new product? 2) What use cases exist, and what is the addressable market? 3) Is there an effective product development process? How competent is the product development team? 4) What is the time frame between ideation and launch? 5) How efficient is the operational process? 6) Is the product prototype tested before or after launch? 7) Are there strategies for mitigating risks emerging from new financial product development? 8) What are the feedback channels for customers' opinions on new products? 9) How does management respond to unfavourable feedback on new financial products?

## 7 Managerial implications

The literature (Schilke, 2014; Pavlou and El Sawy, 2011) provides empirical evidence that suggests that the more an organization invests in product development practices, the higher the likelihood of product success. Effective product development practices (PDPs) serve as forms of competitive strategies, especially in an industry with multiple players (Cheng and Yeng, 2019). DC theory argues for the development of capabilities that help a firm meet the demands of a dynamic environment of business. In the Nigerian financial sector, management teams of FSPs must build the capabilities required for product development to ensure the high performance of their products. It should be noted that executive commitment and support are key success factors for both product development teams and ultimate product performance.

### 7.1 Study limitations and directions for further studies

A limitation of this study is the limited number of respondents, which makes the generalization of the findings challenging. Moreover, the busy nature of the category of respondents (being c-level executives) meant that some were unavailable, and single interviews were sometimes conducted twice to enable the authors to gather rich data. This reduced the number of respondents for some FSPs to one, making it difficult to achieve diversity across functional roles in the product development spectrum and, to a certain extent, leading to potential data loss. Further studies should aim to reach a larger sample size and involve several respondents in a single organization to achieve a higher level of saturation. Future studies can further distil the issues into distinct FSP types such as banks, insurance companies and pension providers in the Nigerian market.

## Author statement

All persons who meet authorship criteria are listed as authors, and all authors certify that they have participated sufficiently in the work to take public responsibility for the content, including participation in the concept, design, analysis, writing, or revision of the manuscript. Furthermore, each author certifies that this material or similar material has not been and will not be submitted to or published in any other publication before its appearance in the *Technological Forecasting and Social Change*.

## Appendix Supplementary materials

Image, application 1
