# Supplementary material for: The role of product development practices on new product performance: Evidence from Nigeria's financial services providers
Source: Technol Forecast Soc Change. 2021 Mar;164:120470. doi: 10.1016/j.techfore.2020.120470 (PMC7893682; doi:10.1016/j.techfore.2020.120470)
Supplement: Supplementary file 1 [file mmc1.pdf]

ELSEVIER

# Certificate of Elsevier Language Editing Services

The following article was edited by Elsevier Language Editing Services:

**"THE ROLE OF PRODUCT DEVELOPMENT PRACTICES ON NEW PRODUCT PERFORMANCE: EVIDENCE FROM NIGERIA'S FINANCIAL SERVICES PROVIDERS"**

Authored by:

**NKEMDILIM IHEANACHOR**

Date: 30-Oct-2020

Serial number: LE-199185-7DDC70C9E336
